# Bibliographical Notices

**Published:** 1851-08

**Authors:** 


### BIBLIOGRAPHICAL NOTICES.

---

*Principles of Physiology, General and Comparative. With three hundred and twenty-one wood engravings.* By WILLIAM B. CARPENTER, F. R. S., F. G. S., Examiner in Physiology and Comparative Anatomy in the University of London, Professor of Medical Jurisprudence in University College, &c. &c. *Third Edition.* 8vo. pp. 1098. Blanchard & Lea. Philadelphia: 1851.

It is pleasing to observe the degree of attention, that is now paid to a department of medical science, which is most assuredly the great foundation for all pathological reasoning. The day has passed when it was necessary to urge the claims of physiology to the attention of every physician. So intimately is it associated with histology, that, although the latter is undoubtedly a branch of anatomy, being nothing more than the minute anatomy of the tissues, it is customary to develope it to a greater extent in treatises on physiology than in those of anatomy. Of late, indeed, we have had excellent works—those of Gerlach, Hassall and others, for example, and the admirable unfinished treatise on Microscopical Anatomy by Kölliker—which are specially devoted to histology, and have added greatly to our knowledge in this direction. Anatomy is far from what it was at the beginning of this century, at the period when Bichat closed his distinguished career. The anatomy of the tissues was cultivated under the name of General Anatomy, but the microscrope had not then rendered such essential service; and organic chemistry, which has shed so much light on histology, was altogether in its infancy. Now, it has experienced such development, that to become acquainted with the new terms which have been introduced to designate successive discoveries, is a work of Herculean—almost hopeless—labor. What would the Cavendishes and the Lavoisiers understand by Allantoin, Alloxan, Parabanic acid, Thionuric acid, Uramile, Alloxantine, Murexide and Murexan? And these only so many results of the oxidation of uric acid. The boundaries of science in all its departments have, indeed, since their time, been largely extended. It is astounding to reflect on the wonderful discoveries which have burst upon us within the present century. Physical science in no like space of time has been so much illustrated; and it has always happened, that the medical sciences have advanced along with it, but not always *passibis æquis.* That their progress should not be to the same degree is not to be wondered at; for even were we to admit, with many recent speculatists, that inorganic and organized bodies differ rather in degree than in kind, the number of efficient causes in constant operation in the latter is such as to prevent us by any mode of calculation from attaining the same certainty in our deductions, and in our estimation of the future, that we arrive at with so much surety in regard to physical phenomena. Even in certain matters that are purely physical we notice this difficulty. Undoubtedly we ought, provided we were possessed of the requisite data, to be able to prognosticate what would be the condition of the heavens as to sunshine or obscuration, at any hour to-morrow, with the same certainty as we can predict the hour at which the sun and moon will rise or set, or an eclipse obscure the face of those luminaries. Thus far, however, the data have not been attainable, and we have, at the present day, as much uncertainty in our atmospheric prognostics as in those that apply to organized bodies—animal or vegetable.

On former occasions we have noticed the enthusiasm that prevailed on the promulgation of the cell-doctrine of the formation of the tissues;—how it was believed by visionaries, that we might now obtain a knowledge of life itself; nay, that we had already arrived at such knowledge. More wise reasoners saw at once, that the "doctrine" carried us but little, or not at all, in advance of our predecessors; and when it was announced as a physiological fact, that all tissues are built up from cells, it still remained to be shown, why those cells arranged themselves according to a definite plan, so that if we were acquainted with the origin of the parent cell, we could foretell as certainly the tissue that would be formed by it, as by knowing the animal from which an ovum had proceeded, we could say what would be the shape, size and other characters of the new being into which it would be developed. The nature of the impulse seated in the primary cell, which gives occasion to such development, has in no wise been elucidated by the discoveries of the histologist and chemist; nor have we any confidence in our ever being able to penetrate the mystery much farther.

It is not strange, however, that this apparently close approximation to the origin—if it may be so termed—of life, as exhibited in the nucleated cell, should have induced the reflecting physiologist to turn his attention more closely to what may be termed the metaphysics of biology, as exhibited in the manifestations of what Von Reichenbach terms the *biod*—the living principle or force, in other words; and to inquire into the relations between it and the physical forces. This has been ably done by the author of the work before us. As an observer every way worthy of credit; as a reasoner of high character on the results of observation, he is worthy of all commendation. Devoted to biological and physical investigations, his best energies have been directed to them; and the result has been a series of works, which have been most favorably received by the profession. "The Principles of Physiology, general and comparative," now appears in its third edition. The first edition was issued about twelve years ago, and was the precursor of two works, more extensively known in this country, owing to their having been reprinted here,—the "Principles of Human Physiology," and the "Manual of Human Physiology;" and of a third, the "Animal Physiology," which has not been re-printed. In the volume before us he has entered into the doctrine of the mutual connection of the vital forces, and their relation to the physical; a doctrine which—he properly remarks—is more fully developed in a paper, by him, on the "Mutual Relations of the Vital and Physical Forces," in the Philosophical Transactions for 1850.

The first section of the third chapter, of the work before us, is 'Of life, or vital activity.' In it the author properly remarks, that "the laws of vital phenomena are as open to investigation as those which comprehend the phenomena of gravity, electricity, or chemical affinity;" yet he transcends our expectations when he adds, "although the intricacy of the combinations, under which these phenomena are usually presented to our observation, renders their laws more difficult of attainment; the success which has attended the philosophical method of inquiry, of late pursued by scientific physiologists, is a most satisfactory proof that they are not beyond the reach of persevering and well-directed research." Although familiar, we believe, with every thing that has been done, of late, by the "scientific physiologist," we confess we have not been led to the same same sanguine inferences as Dr. Carpenter. His views will be best shown by the following extracts:

"Another argument for the mutual relation here contended for, is furnished by the analogy of the Forces operating in the Inorganic world. Reasons have been lately given* for the belief, that Physical Forces of every kind,—namely, Heat, Light, Electricity, Magnetism, Chemical Affinity, and that which produces or resists Motion,—are all 'correlative,' or have a relation of mutual dependence; each being capable of producing any one of the rest, either directly or through the medium of some other; and each being consequeutly producible by any of the remainder. And we may observe that this conversion of one force into another is due to its passage through some material body. Thus the Motion of a body, retarded or resisted by friction, gives rise to Heat; and conversely, heat applied to any form of matter produces its expansion, *i. e.* Motion. The friction of two dissimilar bodies produces not merely heat but Electricity; and Heat itself, when made to act on certain combinations of metals, also produces Electricity; whilst, on the other hand, the Electric current may produce Heat, Light, Magnetism, or Motion, according to the nature of the substances through which it is transmitted. Light, Heat, and Electricity, again, are closely related to Chemical Affinity, which is often specially excited by them (§ 68), and which can, in its turn, generate these Forces; a material *substratum* being required in both cases. So in the beautiful experiments by which Faraday has brought Magnetism to act upon Light, the effect is only produced when the ray is being transmitted through certain material substances.

"Now, the relation which exists among the several endowments peculiar to Organized Structures, and therefore distinguished as *Vital*, appears to be most appropriately expressed in the very same form;—they may be said to be mutually *correlated*, in such a manner that each

* See Prof. Grove on the "Correlation of Physical Forces," 1846.

can either directly or immediately produce the other. The most universal and characteristic of these forces has been shown to be that from which 'Cell-formation' proceeds; and out of this arise all the other agencies. Thus, the tissue of the Muscle is constructed solely with a view to its manifestation of contractile power; and the development of Nervous matter has reference entirely to the peculiar agencies in which it is to be concerned. The contraction of a Muscle, and the production and transmission of a Nervous impression, are phenomena of a distinctly vital character; and it will hereafter appear that, with the manifestation of the peculiar attributes of these tissues, they cease to maintain their existence as living structures,—as if the general Vital force which they previously possessed, had been expended by its conversion into the Muscular and Nervous powers. Here, too, we see that a certain material *substratum* is requisite for the conversion of one force into another; the vital force only being able to produce contractile action, by means of a certain variety of organized structure, which we term Muscle; whilst it can only develope itself as nervous agency, through that peculiar form of tissue which is called Nerve. Again, we find that the nervous force can evoke the muscular, and this in a degree proportional to the intensity of its own excitement; and nervous agency would seem capable of influencing cell-formation, in such a manner as to justify the idea that it is actually re-converted into the form of vital force concerned in it.

"If this view be considered to have a certain claim to reception as (to say the least) a probable hypothesis of the mutual relation of the various forms of Vital activity, we have next to consider whether any similar 'correlation' can be traced between the Vital and the Physical forces; since, if such can be shown to exist, the probability of the hypothesis will be greatly increased. Now, it will be shown (Sect. 3) that all Vital activity is dependent upon the constant agency of one, at least, of the Physical Forces already enumerated—viz. Heat; and that Light and Electricity, operating through Chemical Affinity, are also concerned in sustaining particular forms of it. These agents, indeed, which have long been known under the tern 'Vital Stimuli,' may perhaps be more justly considered as the real Forces concerned in the production of Vital phenomena, modified in their operation by the organized structure through which they are exerted; just as Heat becomes Electricity when it passes through a certain combination of metals. And this idea is borne out by the fact that the Vital Forces appear capable of being in their turn reconverted into the Physical; as in those numerous cases already referred to (§ 50), in which the whole energy of one set of cells is directed to the performance of some *chemical* transformation, and that of another to the production of *mechanical* movement; as well as to other instances, in which it seems nearly certain that the liberation of Electricity, and perhaps also of Heat and Light, are due to Nervous power operating through a particular structure (chap. xvii.). Indeed, when we come to consider the manifestations of Nervous agency in more detail, we shall find that it is at least as closely correlated to Electricity, as Electricity is to Light or Heat (§ 246) ; and the intimacy of this relationship, existing between one of the most special or peculiar of all the Vital forces and one of an exclusively Physical nature, strongly confirms the idea just expressed in regard to the mutual connection of the remainder."

All these views are ingeniously conceived, and ably expounded; yet, do they advance, in any degree, our knowledge of the intricate agency concerned in the production of the vital manifestations? The mystery of the development of the oak from the seed; and of the animal of definite size, shape, and organization from the primordial cell, remains as enigmatical as ever; and when we speak of an impulse or T r i e b seated in the ultimate nucleus of a cell, which gives occasion to cell-formation, we but employ an expression to veil our ignorance.

It is impracticable for us to give an analysis of the varied contents of this most useful volume. Its production has been a labor of love with the author; and has subjected him to much thought, and to no little toil, without the expectation of pecuniary profit. It is to be hoped, however, that so much ability, zeal and industry may meet with their reward, and that future editions may remunerate him for productive exertions, so beneficial in their results to others. It is easy for the superficial and the malevolent to carp at those who are more distinguished than they; and to animadvert on works which, like the present, profess to give a comprehensive view of the existing condition of a confessedly difficult department of science, on which numerous observers have already communicated to the world the results of their observation and reflection. Coincidence of sentiment must often be apparent; and any treatise on such a subject would be most imperfect, which should be restricted to the promulgation of the sentiments of the author, however eminent and capable he might be.

"In the selection of his materials," says Dr. Carpenter, "the Author has endeavored to avail himself of the best and most recent information he could procure upon each department of the subject; it is scarcely to be expected, however, that he should be equally well-informed upon every point; and those who have followed particular departments into detail, will doubtless find scope for criticism in what they may regard as deficiencies, or even as errors. Here, again, the Author must beg that his work may be estimated by its *general* merits; and rather by what it *does*, than by what it *does not* contain. It would have been far easier to expand it by mere compilation to twice its present dimensions, than it has been found to press the collected materials within the space they even now occupy.

"It has been the Author's endeavor, wherever practicable, to draw the materials, both for his text and its illustrations, *direct* from original Treatises and Monographs; and thus to avoid the errors which too frequently arise from second-hand transmission. To have attempted, however, to assign each individual fact to its original discoverer, each doctrine to its first enunciator, would have at least doubled the bulk of a volume which has already far outgrown the dimensions originally intended; and while most desirous to avoid taking credit for what is not his own, the Author has felt himself compelled to limit his references, for the most part, to those *new* facts and doctrines, which cannot be yet said to have become part of the common stock of Physiological Science. The Illustrations not his own are referred to their originals, in the list at the commencement of the volume; and this list, together with the occasional foot-notes, will serve to indicate the principal sources on which the Author has relied for his information, as to those points into which he has not personally examined. From the mode in which these materials are combined and arranged, he believes that he has frequently been able to develope relations between facts and phenomena, which, in the isolated form in which they were originally stated, had only that value which all statements of truth possess; and that he has thus been able to impart to them a new and unexpected significance.

The Author thinks it but due to himself to state, that as the almost constant occupation of his time in other indispensable avocations, leaves him much less opportunity than he would desire for the prosecution of those original researches in which he feels the greatest satisfaction, so he trusts that the same plea may in some degree avail him, in regard to any deficiencies which may be found in the present work. The whole of his disposable time has been devoted to it for nearly two years, not merely without the anticipation of the slightest pecuniary reward, but under the certain loss involved in the relinquishment of other literary engagements of a remunerative character; and the sale of the entire edition will not do more than remunerate his liberal Publisher, for the very large outlay which has been incurred in the production of the work, and more especially for the beautiful series of illustrations with which it is embellished, most of them having been executed expressly for it by Mr. Bagg."

We may remark, in conclusion, that the work is beautifully gotten up in the English fashion, and that the illustrations are in the first style of art.

*Spectacles; their uses and abuses in long and short-sightedness, and the pathological conditions resulting from their irrational employment.* By J. SICHEL, M. D., of the Faculties of Paris and Berlin. Translated by H. W. WILLIAMS, M. D. Boston, Phillips, Samson and Co., 1850.

Mr. Sichel's work is now before the public, medical and non-medical, of the United States. We say non-medical, because we think that the work in question was intended by the author for the use of the people at large—to warn them of the extreme folly and danger of straining and destroying so all-important an organ as the eye, by delaying the use of appropriate glasses when required, or of commencing their use with those which magnify—a certain proof that they are too strong.

Now many of our non-professional acquaintances fancy they can cure consumptions, catarrhs, liver complaints, indigestions &c., &c., much more rapidly and effectually than men who have devoted their lives to such studies, but they generally admit themselves to be lamentably ignorant on the subject of their own optical apparatus; or of the meaning of the terms, Myopia, Presbyopia, Amblyopia, Kopiopia, and the like ; nor can we blame them ; for though such eccentricities are useful in their way, yet we think that a man enjoying any other mode of earning his bread, would be badly employed in studying them. Nor can we refrain from the expression of the opinion, that if M. Sichel did mean his book to be useful and popular with the masses, which we cannot doubt, he would better have served his purpose by simplifying his terms, so that all could have read and understood without difficulty a work in other respects perfectly free from anything bordering upon the affectation of learning,—a work full of sound philosophy, simply and clearly expressed, and full of information which every man ought to possess ; for we truly believe with M. S. that even a small amount of information on the subject, by the poorer classes of society, such as mechanics, seamstresses, &c., would be the means of preventing a vast amount of uneasiness and suffering.

The work in question commences with some general remarks upon the different compass of vision in different subjects—an explanation, with the derivations of, the words Myopia and Presbyopia, a statement which every body knows to be true, that the point of normal vision cannot be established with precision, because it varies infinitely in different individuals, and not only in different, but in the same individual under different circumstances, all of which is, we believe, fully admitted.

The following quotation from the author upon the subject of far and near-sightedness, is a good specimen of the manner in which he treats his subject.

"The sight of man, in a natural state, has generally a somewhat extended range; we might even say that the greater number are born with long sight, and that in his primitive condition, man is presbyopic. All travellers cite more or less astonishing instances of the extent of the power of this faculty in most savage tribes; some even deny their capability of discerning small objects placed near."

We perfectly agree with the author in considering this an absurd exaggeration; for though a savage may be chiefly employed in hunting, and his eye from habit acquire a vast power of traversing distances, yet every body in this country is perfectly aware of the equally extraordinary power of minute observation displayed by the American Indian in the pursuit of his game, or in following up the trail of an enemy. M. Sichel proceeds as follows:

"Among civilized nations primitive long-sightedness is also the general rule; and when particular tastes as, that for hunting, for travelling, especially by sea voyages solicit the frequent employment of this sense, it soon acquires a very great power. Among those who reside in the country, and devote themselves to agricultural pursuits, short sightedness is rare.

"When on the other hand, by inclination or necessity, the sight is assiduously, and from early life, exercised upon small and near objects as is almost always the case in our state of civilization, the sight forced to accommodate itself to these distances, becomes very early shortened. Even before the infant can walk, objects are given him for playthings, which he is obliged to bring near his eyes in order to see all their details. A few years later he is placed at school: there sometimes to distinguish the too small print of a book, sometimes to write in a fine character; he is forced to incline forwards towards his paper or his books. Born among the working classes, the child is none the more favored on that account in the education of this sense. As soon as may be he is placed in apprenticeship, and not only is often constrained to exercise his sight upon minute details of form, but the fatigue of his arms forces him to bring the objects yet nearer to his eyes. Habituated continually to adjust his sight to these limited distances, he finally ends necessarily by closing the faculties of accommodation to distant objects, and becomes myopic."

All this is very true, but unless we can change the whole social system, boys must continne to learn to read and write, and eyes to be sacrificed on the shrine of necessity. Indeed, were it otherwise, M. Sichel's hobby, the spectacle, could never have have had an existence.

Upon the causes of Myopia and Presbyopia the author writes as follows:

"The immediate cause of presbytia, is, a too feeble refractive power of the transparent media of the globe of the eye, or a too short antero-posterior diameter of the organ. In these two cases rays coming from a not distant point, and being in consequence too divergent, unite too late, and form their image behind the retina. In order to concentrate them in a normal focus upon the membrane charged with the transmission of the impression to the brain, collective, that is to say convex glasses, of exactly sufficient power to supply the deficiency of refractive power in the eye become necessary. The remote causes of the insufficiency of this power are somewhat numerous; either a too slight curve of the parts constituting the dioptric apparatus, or the too small quantity of the vitreous or aqueous humors, as well as their thinner consistence; or again, an excessive muscular action are capable of destroying the normal convergence of the rays."

"The contrary is true in myopia; the refractive power of the eye is too great, or its antero-posterior axis too long; as a consequence, the rays coming from distant points, which are very slightly divergent and almost parallel, will be brought to a focus in front of the retina, and in order to paint their image upon this membrane, they require the aid of concave glasses, of which the dispersive power should be equal to the excess of refraction."

Every optician in the United States, we presume, is aware of all this, nor does M. S. give it as a novelty. We have merely quoted it as an illustration of the extremely clear and intelligible manner in which he treats the scientific parts of his subject.

The author does not encourage the very commonly existing opinion, that it is possible to tell from the exterior formation of the eye, namely, its rotundity, or flatness, whether a man is far-sighted or near-sighted; on the contrary, he maintains, that it is only by exception, that the exterior conformation of the eye—by an excess or a want of convexity of the cornea and of the globe in general—permits us to form an opinion as to its being myopic or presbyopic. In most cases he says it furnishes no indication in this respect; the internal parts by their disposition and structure alone, determining the compass of sight.

A chapter is devoted to a description of the shape and manufacture of the glasses, appropriate for the use of presbyopes and myopes; another on the size and general formation of the spectacle. The remarks are judicious, and coming from so distinguished a source, are calculated to be of great utility; but as they are subjects which our best opticians are continually studying, we will spare our readers any unnecessary repetition, and husband our own space, which is but limited, for the purpose of making one or two quotations from the more interesting part of the book, viz: the author's general treatment of patients, his special advice with regard to the use of spectacles, and the general results of said advice.

"Every optician knows, that presbytics are constantly obliged by the necessities of our social system to use their eyes at much nearer distances than the natural focus of their vision will, without injurious consequences, admit of. It is true, that if the organ be naturally strong, these injurious consequences will be slow to manifest themselves; but if there be a tendency to congestion or scrofulous conjunctivitis, any over exertion of the eye, particularly at a focus unnatural to its physiological constitution, will make itself felt with fearful rapidity."

The author's advice as to the means to be employed for the avoidance of the consequences that may follow this neglect of the physiological laws of the sense of vision is so judicious, simple and practical, that we will give it as far as our space permits, and as connectedly as possible, in his own words.

"Whilst reading, writing, or engaged in other occupations requiring minute attention, the objects should be removed to the longest possible distance at which their distinctness shall be unimpaired. This distance may easily be determined by moving the object backwards and forwards, and should always be maintained during assiduous employment. During other less pressing occupations they should, on the contrary, exercise their sight at less di tances, in order to preserve the faculty of accommodation, and to accustom themselves to see objects within their normal focus. But at all times they should be careful to interrupt themselves frequently, in order to change their sight to the most distant objects they can perceive. Every presbytic would do well, after five or ten minute's work upon minute, near objects, to raise the eyes and turn them for an instant to the most distant parts of the room, or even, if he be near a window which is not entered by full sunlight, to look out from his apartments and endeavor to distinguish the details of distant objects.—This exercise, by alternately lengthening and abridging the range of vision, preserves the faculty of accommodation and the normal focus, at the same time that it prevents the enfeebling of vision."

An interruption of a few instants is sufficient to ensure all the salutary effect of this exercise. From personal experience (being ourselves slightly presbytic) we can vouch for the usefulness of the author's advice ; some will say that such interruptions are troublesome and frequently impossible, but the cessation from occupation for half a minute or even a shorter time, is sufficient; and the habit once fixed, the interruption is no longer even perceived. The more closely these rules are observed, the better will the eyes resist the effects of time and use, and the later will the necessity for the use of spectacles be felt. The following passage we recommend to the particular attention of parents, and those who have the charge of the education of youth, as we have seen its inculcations constantly neglected, especially in our public schools.

" There are also certain acquirements necessitated by civilization, such as, writing, drawing, reading, without an exercise of the sight in a nearer focus than that normal to the presbytic. It will be fortunate if the bad methods of education of the child do not supervene to cause him to reduce this distance yet further. Visit a school of children who are beginning to write, and a large proportion of them will be found with the nose nearly upon their paper. In all these cases the precautions referred to are of the first necessity. It is very important to insist that children keep objects at the greatest possible distance ; that they sit erect ; and that they should be taught to read and write in very large characters, at least during the first years."

Long observation and repeated experiment, have convinced the author that amblyopia and presbyopia are intimately connected ; in this opinion he differs essentially from MM. Bonnet and Petrequin, who remark, that they have never encountered amblyopia except in the myopic. While Mr. McKenzie accords to the two ranges of vision in this affection nearly an equal frequency.

M. Sichel accounts for the differences of opinion on this point, in the following manner.

"I have been able to determine that the observations recorded, as having been made upon my eyes, were to be ascribed either to acquired myopia, that is to say, presbytia changed into myopia, or to congenital amblyopia with presbytia simulating myopia, or finally, to myopia augmented by the use of too strong or prematurely employed concave glasses. It is only in this last case that I have seen ocular lassitude, amblyopia, and even amaurosis, united with myopia."

The treatment given by M. Sichel for uncomplicated presbytic amblyopia, is extremely simple, viz., rest, the exercise of the sight upon distant objects, in order to re-establish the normal focus.

Absolute repose in more advanced stages of the malady. If the patient continue his labors, he must be directed to keep his work at as great a distance from him as he possibly can; and when the affection is more advanced, use convex glasses selected according to the general rules. In addition to this, he must use lotions of cold water, adding a small quantity of brandy, to render it more stimulating. "The minor degrees of this affection will promptly yield to these means." When it is more advanced, stronger stimulation will be necessary—vesication, strychnia, &c. In severe cases, of course the result will greatly depend upon the habits and circumstances of the patient.

That original long-sightedness should produce short-sightedness seems somewhat paradoxical, but so it is, and thus it is. The presbytic is constantly constraining his eyes to act at a focus unnatural to the organ. This constant violation of physiological laws sooner or later ends in the destruction of the faculty of accommodating his sight to distant objects, and in shortening his natural focus, even to the degree of causing him to contract a secondary myopia. Now, as M. Sichel states, if the patient should under these circumstances, be induced to wear concave glasses, he will be sure to aggravate his condition.

Our author in speaking of this affection, says, that "at the commencement the myopia is rather apparent than real; it is long-sightedness which has been forced to accommodate itself little by little to too short distances. The sight of the patient shortens slowly and insensibly; he feels, or those about him perceive, that he becomes myopic, that he is forced to bring objects nearer and nearer during his work.

"We have already said that a considerable number, and perhaps the great majority of short-sighted persons have become so in this manner. In children especially, this acquired myopia supervenes very frequently, and in most cases slowly, in consequence of the bad habit which they so easily form of leaning forward over their work."

We agree fully with M. Sichel that in this manner myopia is frequently produced, but differ from his position, that these children must have originally been presbyopic. On the contrary, we are convinced that close observation will satisfy the enquirer, that in the majority of such cases, the children were not originally far-sighted, but that their eyes were feeble, either congenitally or as a consequence of some of the diseases incidental to childhood, such as measles, scarlatina, &c. &c., which will often leave behind them some local debility, which common observers will hardly perceive, but which months, nay, even years of care, will sometimes fail to remove. This debility, of course, will induce the child to bring his eyes closer and gradually closer to his work, until, as the author observes, an inveterate myopia is produced.

We give in the following quotation the author's views regarding the treatment of acquired myopia:

"When the malady is neither very ancient nor far advanced, it is sufficient to arrest its progress, or even to create a retrograde impulse, to cause objects to be removed little by little, to greater distances, according to the rules established for the treatment of myopia in general. Work should be very frequently interrupted, and the intervals should be devoted to observations of distant objects, and to bathing the eyes with cold water. This very simple treatment, if early and perseveringly employed, especially if the subject is still young, is sufficient to obtain a complete and radical cure, and to restore the primitive focus of vision."

"Spirituous liniments, excitants, fugitive blisters, &c., are useless so long as the affliction is uncomplicated with enfeebling of vision. The use of concave spectacles should be most rigorously interdicted, they definitively fix the myopia and render it incurable."

The second part of the volume is devoted almost entirely to the consideration of myopia and its complications. This affection, as the author states, may, like presbytia, give rise to amblyopia and amaurosis, and the abuse of concave glasses will lead to unpleasant consequences, even more rapidly than will the improper use of convex ones in presbytia.

This affection is often accompanied by cerebro-ocular irritation, in which case M. Sichel recommends an antiphlogistic derivation—such as leeches to the anus, small bleedings from the foot, with purgatives and dry-cupping upon the lower extremities and lumbar region ; sometimes the local and general use of mercurials with that of preparations of belladonna.

"Too early recourse to exciting liniments and blisters in the neighborhood of the affected organ should particularly be avoided ; it is better, towards the decline of the irritation, to place a large blister between the shoulders, and afterwards transfer it to the back of the neck."

The author recommends the same general hygienic treatment for myopia as for presbytic amblyopia.

We feel that we have already overstepped our limits, therefore must content ourselves with one more quotation.

"It is not to be forgotten that spectacles generally diminish and end by annihilating the power of accommodation; they fix the range of vision at a certain distance from which it cannot vary. It thus results that concave glasses first confirm myopia and then increase it, and increase it the more in proportion as they are stronger."

The symptoms and treatment of several cases are given in the course of the work, which, as they serve to illustrate the author's views, are worthy of attentive perusal. The book is a highly useful one, and may be read with interest by men of all professions. Its great fault, we think, is a too frequent repetition, which, though we can perceive it to be to a certain extent unavoidable with such a subject, makes it somewhat monotonous. The meaning of the author is thoroughly given by the translator, but the book abounds with Gallicisms which might have been avoided with advantage, for though we admire the French language exceedingly, we do not like French English.

The paper and printing are unexceptionable. In this respect, the Boston publisher seems to have entered fully into M. Sichel's feeling on the subject, and humanely considered the eye-sight of the reading community. For ourselves we especially tender him our sincere thanks.

*Surgical Anatomy.* By Joseph Maclise, Surgeon. *With colored plates.* Part IV. Philadelphia, Blanchard & Lea, 1851.

We have already, in notices of previous numbers, so freely and decidedly expressed our favorable opinion of this admirable reprint, that it seems almost "like a thrice told tale," to reiterate it on the appearance of each successive fasciculus.

The unanimous approval of the whole medical press, and the sanction of the teachers of this department in the various schools, have already stamped it as a work of rare merit, and we are happy to see that the present number does not in the least detract from its enviable reputation.

The contents of Part IV. present the dissections and surgical relations of parts of deep interest to the lithotomist; a brief enumeration of them is all that the limited space allotted to such notices will admit of.

Plate 47 exhibits the surgical dissection of the principal blood-vessels and nerves of the iliac and femoral regions. Plates 48 and 49, the relative anatomy of the male pelvic organs. Plates 50 and 51, the surgical dissection of the superficial structures of the male perineum. Plates 52 and 53, the surgical dissection of the deep structures of the male perineum, together with the lateral operation of lithotomy. Plates 54, 55, 56, the surgical dissection of the male bladder and urethra, with a comparison of the lateral and bi-lateral operations of lithotomy. Plates 57 and 58, congenital and pathological deformities of the prepuce and urethra—stricture and mechanical obstructions of the urethra. Plates 59 and 60, the various forms and positions of stricture and other obstructions of the urethra—false passages—enlargement and deformities of the prostate. Plates 61 and 62, deformities of the prostate—deformities and obstructions of the prostatic urethra.

From this brief enumeration of the contents, our readers can judge of the extent of this number. Of its excellent artistical merits, they can only satisfy themselves by personal inspection, from which they cannot fail to rise with the conviction, that both in the plates, and in the judicious and perspicuous commentaries, the surgeon has here offered him a complete and reliable guide. The work will be completed in one more number, which will be published at the price of one dollar.

---

*Urinary Deposits, their diagnosis, pathology, and therapeutical indications.* By GOLDING BIRD, M. D., F. R. S., F. L. S. Assistant Physician and Lecturer on Materia Medica and Therapeutics, at Guy's Hospital, &c. &c. *Second American, from the third and enlarged London edition.* Philadelphia, Blanchard & Lea, 1851.

The new edition of Dr. Bird's work, though not increased in size, has been greatly modified, and much of it re-written, and now presents in a compendious form, the gist of all that is known and reliable in this department. From its terse style and convenient size, it is particularly applicable to the student, to whom we cordially commend it.

